# Preliminary Results of Pilot Qualitative Study of New Wave of Early Career Psychiatrists’ Migration in Europe

**DOI:** 10.1192/j.eurpsy.2026.11632

**Published:** 2026-07-31

**Authors:** M. Spasić Stojaković, S. Tanyeri Kayahan, A. K. Sikora, C. M. Platsa

**Affiliations:** 1 Kliniken im Theodor-Wenzel-Werk, Berlin, Germany; 2 Psychiatry Clinic, Republic of Turkiye Ministry of Health Yalvac State Hospital, Yalvac, Isparta, Türkiye; 3 Mazovian Specialist Hospital “Drewnica”, Ząbki; 4 Department of Psychiatry, Medical University of Warsaw, Warsaw, Poland; 5 National Medical University, Ivano-Frankivsk , Ukraine; 6 Central and North West London NHS Foundation Trust (CNWL), London, United Kingdom

## Abstract

**Introduction:**

Foreign-trained doctors form a substantial part of the UK and EU healthcare workforce. Since 2020, migration in Europe has shifted due to COVID-19, Brexit, the Ukraine war, and rising political oppression. Literature confirms the migration motives have changed, and suggests adding family and safety/security migrants to health worker typologies. Literature on psychiatrist migration focused on intentions rather than experiences and integration. Our study fills this gap by qualitatively examining early-career psychiatrists (ECP) who migrated after 2020.

**Objectives:**

Capturing key aspects of ECP migration experiences using qualitative reflexive thematic analysis, and evaluate the feasibility of the method to this research area.

**Methods:**

We conducted a pilot exploratory qualitative study - reflexive thematic analysis. Participants were alumni of the 2022 European Psychiatric Association (EPA) Summer School, selected via criterion sampling: those who had migrated since 2020 - 7/21 participants, all female; 6 agreed to participate, 5 provided written migration narratives (open-ended essays). Data collection and thematic analysis are ongoing.

**Results:**

Systemic push and personal pull factors were described: an early migration intention formation (residency, studying), with the decision to migrate driven by political factors in the home country for four participants (war, corruption, political oppression, lack of safety), and by career aspirations and poor working conditions for two; three participants migrated accompanying family, and one temporarily. None of the participants had access to EU mobility measures and all faced housing and financial difficulties, along with the complex, costly bureaucracy causing professional degradation, identity loss, and exhaustion. Workplace recognition was the linchpin of integration, reforging professional identity and belonging. Resilience stemmed from core values, stress-relief routines, and supportive relationships, including EPA activities ensuring identity continuity. Migration was viewed as a journey of maturity and integrity, strengthening the confidence, identity, and core values - recognizing challenges as structural, not personal failures, and some learning to embrace rather than resent the process. Family was a multifaceted theme - driving migration, adding strain, yet its thriving signifying successful integration. All were specialists at home; none work as such abroad, yet none regrets migration and would recommend it.

**Conclusions:**

Thematic analysis suited rich data from few participants, providing themes to inform future quantitative or broader qualitative research and guide policy in host and home countries. Larger studies could use snowball sampling to data saturation, framework analysis, and include those with unsuccessful migration. Findings underscore the need for standardized training and the vital support of EPA for its migrant members.

**Disclosure of Interest:**

None Declared

